# Effects of high-pressure-processed rice intake during interval walking training on glycemic control and *NFKB2* gene methylation in hyperglycemic older people

**DOI:** 10.1007/s00394-024-03536-2

**Published:** 2024-11-26

**Authors:** Takamichi Aida, Shizue Masuki, Mayuko Morikawa, Kazumasa Manabe, Mayuka Furihata, Aki Maekawa, Tomoyuki Fujita, Hiroshi Nose

**Affiliations:** 1 Department of Sports Medical Sciences, Shinshu University Graduate School of Medicine, 3-1-1 Asahi, Matsumoto, 390-8621 Japan; 2 Department of e-Health Sciences, Shinshu University Graduate School of Medicine, Matsumoto, Japan; 3 Institute for Biomedical Sciences, Shinshu University, Matsumoto, Japan; 4 Jukunen Taiikudaigaku Research Center, Matsumoto, Japan; 5 Department of Agriculture, Shinshu University Graduate School of Science and Technology, Minami-minowa, Japan

**Keywords:** High-pressure-processed rice, Interval walking training, Glycemic control, *NFKB2* gene methylation, Hyperglycemic older people

## Abstract

**Purpose:**

High-pressure-processed (HPP) rice is white rice that maintains some key functional food ingredients of brown rice, such as polyphenols. We examined whether HPP rice intake during interval walking training (IWT) improved glycemic control with enhanced methylation of the *NFKB2* gene in hyperglycemic older subjects.

**Methods:**

We recruited 51 people aged ~ 70 yr who had already performed IWT for ≥ 6 months, but had hyperglycemia (blood glucose concentration ([Glc]) > 110 mg/dl or HbA1c > 6.0% while fasting). Participants were randomly divided into control (CNT) or HPP rice (HPR) groups and instructed to perform IWT for an additional 4 months while ingesting 75 g dry weight of either white rice or HPP rice, respectively, at every breakfast and dinner. Before and after intervention, [Glc] was measured by continuous glucose monitoring for 5 days, with standardized breakfast on *day 5*. Methylation of *NFKB2* was measured by pyrosequencing.

**Results:**

After intervention, mean fasting [Glc] values for 180 min before breakfast over 4 days (*days 2–5*) marginally decreased in HPR but were not different from CNT (P = 0.17). However, the standard deviation during the period decreased more in HPR than in CNT (P = 0.013). Moreover, total area under the curve (tAUC) for 180 min after breakfast on *day 5* decreased more in HPR than in CNT (P = 0.035). The change in tAUC on *day 5* after the intervention was negatively correlated with that in *NFKB2* gene methylation (P = 0.002).

**Conclusion:**

HPP rice intake during IWT improved glycemic control with suppressed reduction in *NFKB2* gene methylation in hyperglycemic older people.

**Trial registration number and date of registration:**

UMIN000024390; October 13, 2016.

**Supplementary Information:**

The online version contains supplementary material available at 10.1007/s00394-024-03536-2.

## Introduction

Exercise prescription has been recommended to prevent and improve lifestyle-related disease (LSD) symptoms in middle-aged and older people [1, 2]. However, the effects, which are reported to be limited, typically plateau after several months of the prescription [3]. To overcome this limitation, supplemental food products containing important dietary nutrients, as well as antioxidants such as polyphenols, are sometimes recommended [4, 5]. While many such supplemental food products are commercially available, few are stable enough to be ingested on a daily basis in order to accelerate the effects of exercise training.

Non-polished rice (brown rice) contains many such functional food ingredients: dietary fiber, vitamins, antioxidants and other bioactive substances whose habitual intake at daily meals has been known to improve LSD symptoms including hyperglycemia [6]. However, because of a widespread perception among Japanese people that brown rice has poor taste and texture [7], many Japanese prefer to eat polished (white) rice, resulting in the loss of many key nutrients contained in rice bran. To address this dietary problem, Fujita et al. [8] have developed high-pressure-processed (HPP) rice in which the water-soluble nutritional components, except for dietary fiber in the episperm and germ of brown rice, are partially transferred into the inner albumen (white rice) during exposure to high-hydrostatic pressure (987 atmospheric pressure) prior to polishing. Experimentally, they confirmed that 80 ~ 90% a large portion of polyphenols, 50 ~ 60% of the B vitamins, and 2–3 times of γ-amino butyric acid (GABA) in brown rice were successfully transferred or accumulated in HPP rice after polishing [9]. Thus, HPP rice, even though it is polished rice, retains many of the functional food ingredients of brown rice, with a taste and texture similar to that of white rice. However, the effects of HPP rice intake on glycemic control and its mechanisms remain unknown in older people with LSDs.

It has been suggested that chronic inflammation is one of the major drivers of LSDs including hyperglycemia [10] and is partially caused by reactive oxygen species (ROS) generated by decreased mitochondrial function that accompanies aging. In the present study, we postulated that polyphenols in HPP rice would scavenge ROS and thereby suppress chronic inflammation to improve hyperglycemic symptoms. Therefore, we measured blood glucose concentration ([Glc]) by the continuous glucose monitoring (CGM) method, and we also measured methylation of the *NFKB2* gene, a master gene for pro-inflammatory responses, as an index of chronic inflammation in the whole body before and after the intervention. We used the CGM method because research has suggested it may be more sensitive at detecting altered glycemic control by intervention [11].

We hypothesized that HPP rice intake at daily meals for 4 months would suppress the demethylation of the *NFKB2* gene and improve glycemic control in hyperglycemic older people. To examine our hypothesis, we recruited subjects from those who had already performed interval walking training (IWT) for ≥ 6 months [12] yet still showed signs of hyperglycemia. We used this criterion because the effects of IWT on LSD symptoms, including hyperglycemia, typically reach a steady state during the first 6 months of training [3], thus enabling us to minimize any influence of interindividual variation of physical activity and detect mere effects of HPP rice on the symptoms.

## Methods

### Subjects

This study was approved by the Review Board on Human Experiments, Shinshu University School of Medicine (approval no. 3542), and it conformed to the standards set by the Declaration of Helsinki. The protocol was registered with the University Hospital Medical Information Network (UMIN) in Japan (trial registration number: UMIN000024390) on October 13, 2016.

**Fig. 1:**
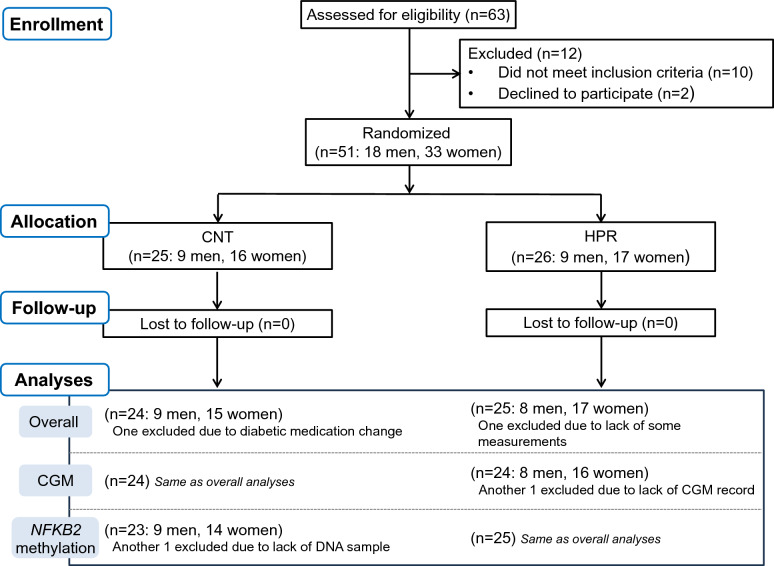
CONSORT flow diagram. *CNT* the control group, *HPR* the high-pressure-processed rice group, *CGM* continuous glucose monitoring. The study was conducted using a randomized, double blind, placebo-controlled design

As shown in Fig. 1, subjects were recruited from participants who had performed IWT for more than 6 months in the “Jukunen Taiikudaigaku Project”, a health promotion program for middle-aged and older people in Matsumoto City, Japan, but yet identified as hyperglycemic (based on a fasting blood glucose concentration ([Glc]_fast_) > 110 mg/dl or HbA1c > 6.0%) when they visited local community offices once a month to receive training instruction.

After the experimental protocol was fully explained to 63 participants, 51 (18 men, 33 women; 56–77 yr) provided written informed consent before participating in the present study.

### Grouping

Subjects were randomly assigned to one of 2 groups, IWT + white rice intake (CNT: 9 men, 16 women) and IWT + HPP rice intake (HPR: 9 men, 17 women), in a 1:1 ratio by an independent investigator (K.H.) using stratified permuted-block randomization (block sizes of 2 to 4). Subjects were stratified according to three factors: gender (male or female), age (> 70 or ≤ 70 years), and baseline HbA1c (> 6.1 or ≤ 6.1%). The random allocation sequence was generated using a computer. We confirmed no significant differences in the baseline measurements (Table 1, Fig. 3A) and in past and current health status and medication usage of subjects between the groups (Supplemental Table 1).

**Table 1:** Physical characteristics, blood constituents, and glycemic control at baseline and changes after intervention

	Baseline		Changes after intervention			Two-way ANOVA [group x time]^b^
	CNT	HPR	CNT	HPR		P value
n	24	25	24	25		
Sex (men: women)	9: 15	8: 17	9: 15	8: 17		
Age, yr	71 ± 5	69 ± 5	NA	NA		–
Height, cm	159 ± 9	158 ± 8	NA	NA		–
Body weight, kg	58.8 ± 2.1	58.8 ± 1.9	0.6 ± 0.3**	0.4 ± 0.2**		0.57
BMI, kg/m^2^	23.3 ± 0.7	23.6 ± 0.6	0.2 ± 0.1**	0.2 ± 0.1**		0.61
SBP, mmHg	146 ± 4	147 ± 3	5 ± 2*	3 ± 3		0.58
DBP, mmHg	78 ± 2	83 ± 2	3 ± 1	−1 ± 2		0.11
${\text{e}}\!\mathop{\text{V}}\limits^{.}\!{\text{O}}_{{{\text{2peak}}}}$, ml/min	1455 ± 75	1511 ± 73	−11 ± 32	12 ± 32		0.61
HR_peak_, beats/min	146 ± 5	140 ± 3	3 ± 4	3 ± 3		0.99
LDL-C, mg/dl	124 ± 6	123 ± 7	2 ± 5	3 ± 4		0.90
HDL-C, mg/dl	73 ± 5	68 ± 4	0 ± 2	1 ± 1		0.49
TG, mg/dl	113 ± 12	120 ± 13	−10 ± 15	−20 ± 10		0.56
[Glc]_fast_, mg/dl	110 ± 3	112 ± 4	6 ± 2**	2 ± 2		0.59
HbA1c, %	6.0 ± 0.1	6.1 ± 0.1	0.1 ± 0.1	0.0 ± 0.0		0.41
Insulin, μU/ml	6.7 ± 0.7	6.8 ± 0.8	1.1 ± 0.5**	0.8 ± 0.4**		0.67

CGM [Glc]^a^ (average of *days 2*–*5*)						
All day						
Mean, mg/dl	121 ± 3	125 ± 4	2 ± 2	−2 ± 2		0.16
SD, mg/dl	21.2 ± 1.5	22.4 ± 2.0	1.1 ± 1.2	−0.5 ± 0.8		0.27
TIR (70–140 mg/dl), %	80.5 ± 3.1	76.6 ± 4.4	−2.7 ± 2.5	1.8 ± 2.0		0.17
TAR (> 140 mg/dl), %	18.7 ± 3.1	23.0 ± 4.4	3.0 ± 2.5	−1.6 ± 2.0		0.16
TBR (< 70 mg/dl), %	0.8 ± 0.4	0.4 ± 0.2	−0.3 ± 0.6	−0.2 ± 0.2		0.95
Pre-breakfast						
Mean, mg/dl	107 ± 3	110 ± 3	2 ± 2	−2 ± 3		0.17
SD, mg/dl	4.2 ± 0.4	5.2 ± 0.5	0.6 ± 0.5	−1.1 ± 0.5#		0.013
Post-breakfast						
tAUC, mg/dl × 180 min	4831 ± 161	5009 ± 215	287 ± 122*	−104 ± 73##		0.008

Values are the mean ± standard deviation (SD) for age and height and the mean ± standard error (SE) for other variables

*CNT* the white rice group, *HPR* the high-pressure-processed rice group, *BMI* body mass index, *SBP* systolic blood pressure, *DBP* diastolic blood pressure, $e\mathop V\limits^{.} O_{2peak}$ estimated peak aerobic capacity for walking, *HR*_*peak*_ peak heart rate at ${\text{e}}\!\mathop{\text{V}}\limits^{.}\!{\text{O}}_{{{\text{2peak}}}}$, *LDL-C* low-density lipoprotein cholesterol, *HDL-C* high-density lipoprotein cholesterol, *TG* triglycerides, *[Glc]*_*fast*_ fasting blood glucose concentration, *HbA1c* hemoglobin A1c, *CGM [Glc]* blood glucose concentration measured by continuous glucose monitoring, *TIR* time in range, *TAR* time above range, *TBR* time below range, *tAUC* total area under the curve, *NA* not applicable

^a^CGM [Glc] data were presented for 24 subjects in the HPR group

^b^Interactive effect of group x time (before vs. after intervention)

Significant differences from pre-intervention value, *P < 0.05 and **P < 0.01. Significant differences from the corresponding values in the CNT group, #P < 0.05 and ##P < 0.01

### Protocol

**Fig. 2:**
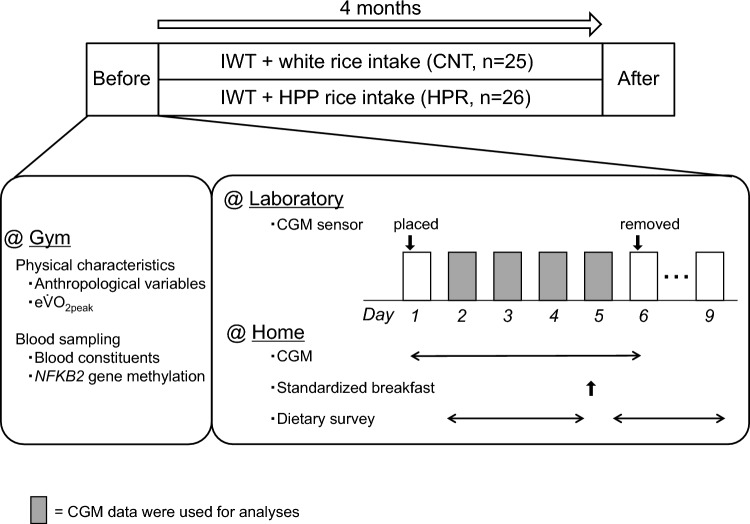
Experimental protocol. *Before* before-intervention assessment, *IWT* interval walking training, *CNT* the control group, *HPR* the high-pressure-processed (HPP) rice group, *After* after-intervention assessment, $e\dot{V}O_{2peak}$ estimated peak aerobic capacity for walking, *CGM* continuous glucose monitoring. Subjects were instructed to perform IWT for 4 months while ingesting 75 g dry weight of test rice at every breakfast and dinner. They were also instructed to ingest a standardized breakfast on the 5th day of the CGM measurement

This study was carried out in a randomized, double blind, placebo-controlled manner. Figure 2 shows the protocol of the present study. The experiments were conducted from October 13, 2016, to May 1, 2017, and from October 4, 2017, to April 23, 2018. For the measurements of baseline physical characteristics, subjects arrived at a gym at 9:00 AM on a day assigned to each subject, and we confirmed that they had refrained from vigorous exercise and did not eat any food except drinking water after 10:00 PM on the day before, as instructed in advance. After measuring the subjects’ anthropological variables, we sampled peripheral blood from the antecubital vein, which was used to determine blood constituents and methylation of the *NFKB2* gene later. Subjects were then allowed to eat a light breakfast and rest for 1 h before undergoing measurement of estimated peak aerobic capacity (${\text{e}}\!\mathop{\text{V}}\limits^{.}\!{\text{O}}_{{{\text{2peak}}}}$) by graded walking test, as described below.

On a separate day assigned to each subject, they reported to our laboratory at 8:00 AM to have their arterial blood pressure measured and to have a glucose enzyme electrode for the glucose monitor placed in their abdominal subcutaneous tissue. They then underwent free-living CGM for 5 days. On the morning of *day 6*, they reported to our laboratory again, this time to have the electrode removed and to have their records for 5 full days transferred to a PC. During the 5 days of CGM, subjects were generally allowed to take food and medicine as before, but a standardized breakfast was given on *day 5* for the precise assessment of [Glc] response by CGM in the morning.

After the baseline measurements and grouping, they performed IWT for the next 4 months as before. The intervention period was determined according to the periods for which we had examined the combined effects of IWT and milk product/dried tofu intake on physical fitness, glycemic control, and pro-inflammatory gene methylation [13–16] so that we could compare the results among the studies. During the intervention, subjects in the CNT group ingested 75 g dry weight of white rice for every breakfast and dinner, respectively, and similarly, those in the HPR group ingested a like amount of HPP rice. If subjects could not eat the rice for testing as scheduled for some reason, they were instructed to eat 150 g of the rice in total per day. The amount of testing rice per day was determined so that it matched the amount that active Japanese of the same age regularly ate per day according to a national nutrition survey in Japan [17, 18].

Subjects received notice before the intervention that appearance and taste of the white or HPP rice might be slightly different from those of rice that they regularly cooked and ate at home since the testing rice was pre-packaged for the present intervention (see below for details). Furthermore, subjects had no chance to directly compare appearance and taste differences between the white and HPP rice used for testing. Accordingly, no subjects reported being aware of which group they were assigned to.

To ensure that subjects continued IWT during the intervention, we used a remotely supervised, internet-based exercise prescription system which monitored exercise intensity during IWT with an original portable calorimeter, transferred the walking records in the device to the server, and provided training instruction from the server [19–21]. The system enabled us to examine the effects of HPP rice by confirming no significant differences in exercise intensity and volume during the intervention between the CNT and HPR groups (Table 2).

**Table 2:** Training achievements over 4 months

	CNT	HPR
n	23	25
Sex (men: women)	9: 14	8: 17
Walking days per week	4.1 ± 0.3	4.2 ± 0.3
Fast walking time per week, min	79 ± 10	75 ± 9
Fast walking		
Time, min/walking day	18 ± 1	17 ± 2
Energy expenditure, mlO_2_/kg/walking day^a^	313 ± 23	294 ± 34
Intensity, mlO_2_/kg/min^a^	17.4 ± 0.7	16.6 ± 1.0
Slow walking		
Time, min/walking day	41 ± 7	37 ± 6
Energy expenditure, mlO_2_/kg/walking day^a^	316 ± 35	305 ± 35
Intensity, mlO_2_/kg/min^a^	8.7 ± 0.6	8.8 ± 0.6

Values are the mean ± SE. We analyzed 23 subjects in the CNT group since we failed in the measurement for one woman

^a^Resting oxygen consumption is not included

After the 4-month intervention, we measured the same variables using the same protocol as before while having subjects continue IWT and the test rice intake, except for breakfast on the 5th day of CGM. We also recorded the average atmospheric temperature (−6.7 to 19.0 °C) and relative humidity (35 to 93%) during the intervention.

### IWT regimen

Subjects were instructed to continue IWT: repeating ≥ 5 sets of fast and slow walking, ≥ 70% and ~ 40% of ${\text{e}}\!\mathop{\text{V}}\limits^{.}\!{\text{O}}_{{{\text{2peak}}}}$, respectively, for 3 min each per day, ≥ 4 days/week [12]. Energy expenditure during the training was monitored with a portable calorimeter (JD Mate; Kissei Comtec, Matsumoto, Japan) carried on the midclavicular line of the right or the left side of the waist. A beeping signal alerted subjects when a change of intensity was scheduled, and a melody notified them when their walking intensity had reached the target level. Since the calorimeter was equipped with a tri-axial accelerometer and a barometer, we could estimate energy expenditure during IWT accurately even if subjects walked on inclines [19]. Subjects visited a local community office near their homes once a month to transfer their walking records in the calorimeter to the server at the administrative center through the internet for automatic analysis and reporting. The trainers used these reports to track subjects’ daily training achievements, and based on these records, they instructed subjects on how best to achieve their target levels. The target intensity for fast walking was not re-adjusted during the 4-month intervention period.

### Rice for testing

Individual servings of white or HPP rice were packaged into small plastic containers, pre-cooked under aseptic conditions and sealed. When subjects ate the rice, they warmed up a container using a microwave oven. As shown in Table 3, the nutritional components of white and HPP rice are almost similar except for threefold more polyphenols and several-fold more GABA in HPP rice than in white rice. The detailed differences in phenolic acid and free amino acid contents between white rice and HPP rice are shown in Supplemental Table 2. Despite the differences in polyphenols and free amino acids between white rice and HPP rice, their appearance and taste were similar.

**Table 3:** Nutritional components of the cooked test rice per daily intake

	White rice	HPP rice
Energy, kcal	523	523
Protein, g	8	8
Fat, g	2	2
Carbohydrate, g	119	118
Total phenolic content, FAE mg^a^	16	49
GABA, mg^a^	0.6	10.3

The values show the amounts of the nutritional components of cooked test rice per day (150 g dry weight before cooked) which was pre-packaged into individual servings. Subjects were instructed to consume 75 g dry weight of either white rice or high-pressure-processed (HPP) rice at every breakfast and dinner for 4 months. *FAE* ferulic acid equivalent, *GABA* γ-amino butyric acid

^a^Composition of phenolic acids and other free amino acids are shown in Supplemental Table 2

The pre-packaged rice for testing was distributed to subjects in advance by a member of this project (N.M. from Asahimatsu Foods, Co., Ltd.) who took no part in the data acquisition, analyses, and interpretation. The containers for the two types of rice for testing were simply coded as A or B so that neither the subjects nor the investigators were aware of the contents until completion of the analyses.

If subjects in either group wanted to eat more, they were allowed to eat white rice cooked by themselves at home. Subjects were instructed to refrain from habitual intake of supplements or functional foods containing nutrients specific to HPP rice in order to avoid their influence on the results.

### Day 5 standardized breakfast

We prepared salt-adjusted side dishes (Miwa Corporation, Okayama, Japan) and pre-packaged white rice (men: Toyo Suisan, Tokyo, Japan; women: Table Mark, Tokyo) as the standardized breakfast on the 5th day and distributed the food to subjects in advance. The nutritional components of the breakfast were 619 kcal (97.8 g carbohydrate, 22.9 g protein, 15.1 g fat) for men and 522 kcal (75.5 g carbohydrate, 22.1 g protein, 14.6 g fat) for women. We instructed subjects to eat breakfast around 8:00 AM and refrain from ingesting any other foods for the following 180 min and from vigorous exercise throughout the day.

### Dietary intake survey

As shown in Fig. 2, a dietary survey was conducted for 7 days: 3 days during the periods for [Glc] measurements by CGM (*days 2–4*, but not *day 5*) and the following 4 days (*days 6–9*). Subjects answered a questionnaire (FFQg Ver 3.5; Kenpakusya, Tokyo, Japan) administered by a dietitian. The nutritional components and their amounts ingested per day are shown in Supplemental Table 3. We confirmed that these intake values generally met the recommended dietary allowances (RDA) for active, older Japanese, except for the relatively high sodium intake [18].

### Measurements

#### ${\text{e}}\!\mathop{\text{V}}\limits^{.}\!{\text{O}}_{{{\text{2peak}}}}$

After baseline measurements at rest for 3 min, subjects walked for 3 min on a flat floor at 3 graded subjective velocities (slow, moderate, and fast speeds) for 3 min each, during which 3-dimensional accelerations were measured at 10 ms intervals and recorded as 5-s memories as averaged values with a calorimeter (JD Mate; Kissei Comtec, Matsumoto, Japan) carried on the midclavicular line of the right or the left side of the waist [19]. ${\text{e}}\!\mathop{\text{V}}\limits^{.}\!{\text{O}}_{{{\text{2peak}}}}$ value was adopted as an average value during the last 30 s at maximal walking velocity. Heart rate (HR) was simultaneously measured with a near-infrared ear pickup probe, and peak HR was adopted as that at ${\text{e}}\!\mathop{\text{V}}\limits^{.}\!{\text{O}}_{{{\text{2peak}}}}$. Regarding the precision of the method, we have confirmed that the ${\text{e}}\!\mathop{\text{V}}\limits^{.}\!{\text{O}}_{{{\text{2peak}}}}$ is highly correlated with $\mathop {\text{V}}\limits^{.} {\text{O}}_{{{\text{2peak}}}}$ obtained by a standard graded cycling test with respiratory gas analysis (r = 0.91, P < 0.0001) [12, 21].

#### Blood samples

The blood sampled from the antecubital vein during the measurements of physical characteristics before and after the intervention was used to determine [Glc]_fast_, as well as fasting blood concentrations of HbA1c, insulin, cholesterol, and triglycerides. The blood was also used to determine methylation of the *NFKB2* gene.

#### CGM

The CGM method (Medtronic iPro®2; Medtronic MiniMed, Northridge, CA) was used to measure [Glc] every 5 min, based on the assumption that [Glc] measured with an enzyme electrode in the interstitial space of subcutaneous tissues in the lower abdominal area is similar to that in blood [22]. To calibrate the electrode sensitivity, we asked subjects to sample a drop of blood from the tip of a digital finger (Fine Touch; Terumo, Tokyo, Japan) and measure [Glc] (Medisafe Fit; Terumo, Tokyo, Japan) 4 times a day: before breakfast, lunch, dinner, and bed. We also asked subjects to record the times when they started eating breakfast, lunch, and dinner.

#### DNA methylation

DNA methylation was determined by pyrosequencing (PyroMark Q24ID; Qiagen, Hilden, Germany). The primers for PCR and sequencing were designed using PyroMark Assay Design 2.0 software (Qiagen). The promoter region of the *NFKB2* gene (−1281 to −1018 upstream of the transcription start site) was amplified by PCR. The primers are shown in Table 4. DNA methylation predominantly occurs on cytosines at sites of CpG dinucleotides in mammals. Therefore, the target region of the *NFKB2* gene was 5′-AAAGGGCGCGAGGCGTGACGCACGGAAACGTCA-3 (−1238 to −1206 upstream of the transcription start site). Briefly, after genomic DNA was extracted from the peripheral blood using the MPLC DNA Isol. Kit Large Volume (Roche Diagnostics, Tokyo, Japan), bisulfite conversion of 500 ng of genomic DNA was performed with an EpiTect Buisulfite Kit (Qiagen). Bisulfite-converted DNA was purified and adjusted to 10 ng /µl. The adjusted bisulfite-converted DNA was amplified by PCR with a reverse primer biotinylated at its 5′end using a PyroMark PCR Master Mix Kit (Qiagen). Biotinylated PCR products were immobilized onto streptavidin-coated beads (GE Healthcare, Uppsala, Sweden), and the DNA strands were separated using a denaturation buffer. After washing and neutralization at a PyroMark Q24 Vacuum Workstation, the sequencing primer was annealed to the immobilized strand. DNA methylation was analyzed via highly quantitative bisulfite pyrosequencing with a PyroMark Q24 system (Qiagen). The data were analyzed using PyroMark Q24 software (Qiagen) and the results are shown in Fig. 4 and Supplemental Table 4.

**Table 4:** Primers used for the pyrosequencing assay

Gene	Amplification site	Forward primer (5′ to 3′)	Reverse primer (5′ to 3′)	Sequencing primer (5′ to 3′)^a^
*NFKB2*	−1281 to −1018	GGGTTGGTTGAGTTAGTTTAGAGTTAAAT	Biotin-CCTCCTCCCTCTTTTCTCTTATCC	−1262-AGAGTTAAATTTTTAGTTAATGAA

^a^The sequencing primer was designed to analyze sense DNA

### Analyses

#### The number of subjects for analyses

**Fig. 3:**
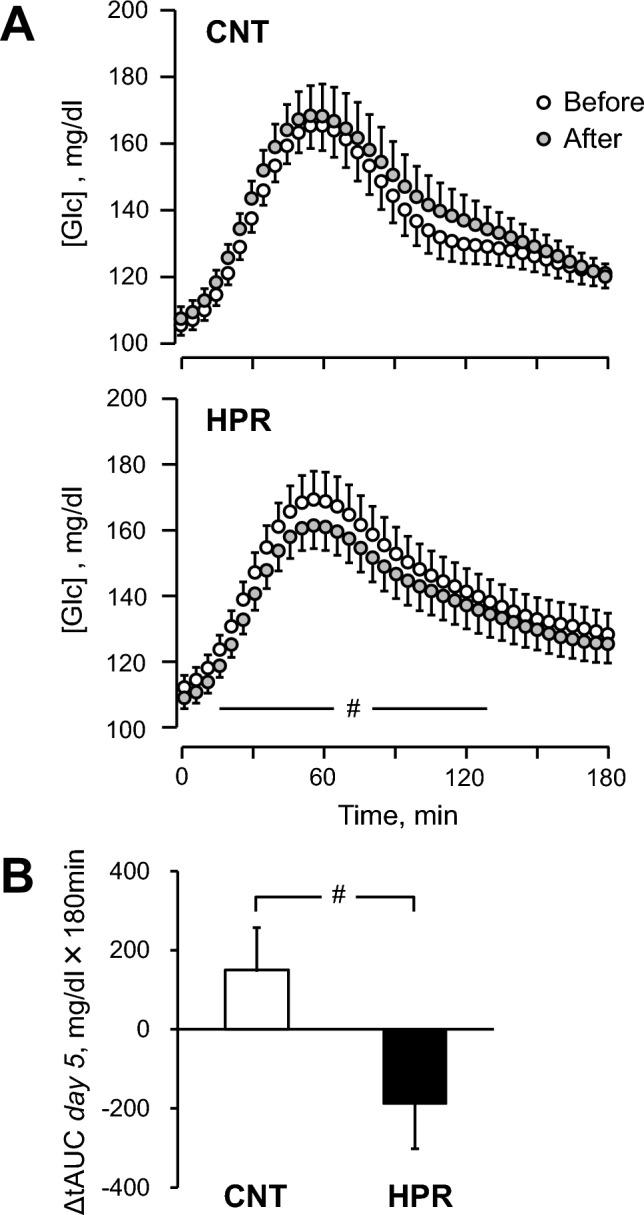
Blood glucose concentration ([Glc]) over 180 min from the start of standardized breakfast intake on *day 5* (**A**) and the changes in post-breakfast [Glc] after the intervention expressed as total area under the curve (tAUC) (**B**). The mean and SE bars are presented for 24 subjects in the CNT and the HPR groups, respectively. For Fig. A, when we examined any significant effects of intervention on a transient [Glc] response to breakfast intake in each group, the two-way ANOVA for repeated measures indicated neither main effect of time (before vs. after the intervention) for the CNT and HPR groups (P = 0.17 and P = 0.11, respectively) nor interactive effect of [time (before vs. after the intervention) x time since starting breakfast intake] for the CNT and HPR groups (both, P > 0.9). On the other hand, when we examined any significant effects of group on changes in the transient [Glc] response after the intervention, the two-way ANOVA for repeated measures indicated significantly greater reductions in the HPR group than in the CNT group (P = 0.034) but no interactive effect of [group x time since starting breakfast intake] (P > 0.9). #Significant differences in the change after the intervention between the groups, P < 0.05

**Fig. 4:**
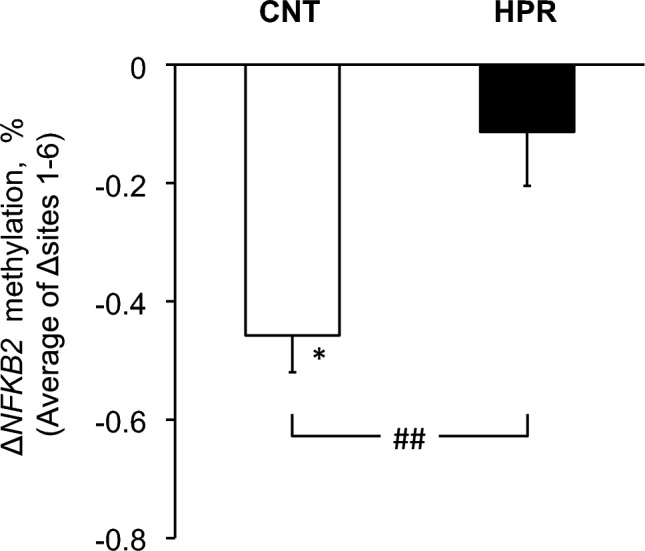
Changes after the intervention in methylation of *NFKB2* gene promoter region measured by pyrosequencing. The mean and SE bars are presented for 23 subjects in the CNT group and 25 subjects in the HPR group. Average changes across CpG sites 1–6 are presented after adjustment by ANCOVA with the pre-intervention values as a covariate. *Significant difference from pre-intervention value, P < 0.05. ##Significant difference between the groups, P < 0.01

Figure 1 (*lower panel*) shows the number of subjects for analyses. As shown in Table 1, we analyzed physical characteristics and blood constituents for 24 subjects in the CNT group and 25 subjects in the HPR group since a woman in the CNT group changed her diabetic medication during the intervention and we failed to obtain some measurements for a man in the HPR group. Moreover, as shown in Table 1 and Fig. 3, we analyzed [Glc] by CGM for 24 subjects in both groups since we failed in the measurement for a woman in the HPR group. Finally, as shown in Fig. 4 and Supplemental Table 4, we analyzed the methylation of the *NFKB2* gene in 23 subjects in the CNT group and 25 subjects in the HPR group since we failed to obtain genomic DNA for a woman in the CNT group

#### Analyses of [Glc] by CGM

We determined overall mean [Glc] and standard deviation (SD) of [Glc] values by CGM on *days 2–5*, as well as percentage of time where [Glc] values on *days 2–5* were in range (70–140 mg/dl, TIR), time above range (> 140 mg/dl, TAR), and time below range (< 70 mg/dl, TBR) [23] and present them in Table 1. Moreover, we determined mean fasting [Glc] and SD of [Glc] values by CGM for 180 min before breakfast on *days 2–5* and present them as pre-breakfast mean and SD in Table 1. This is because we thought that we could precisely measure [Glc] average and SD levels in the fasting condition for more than 8 h after the last dinner. In addition, we analyzed a transient response of [Glc] for 180 min after breakfast on *days 2–5* and expressed it as post-breakfast total area under the curve (tAUC) in Table 1. Furthermore, we separately analyzed a transient response of [Glc] on *day 5* when the standardized breakfast was given (Fig. 3).

### Statistics

**Fig. 5:**
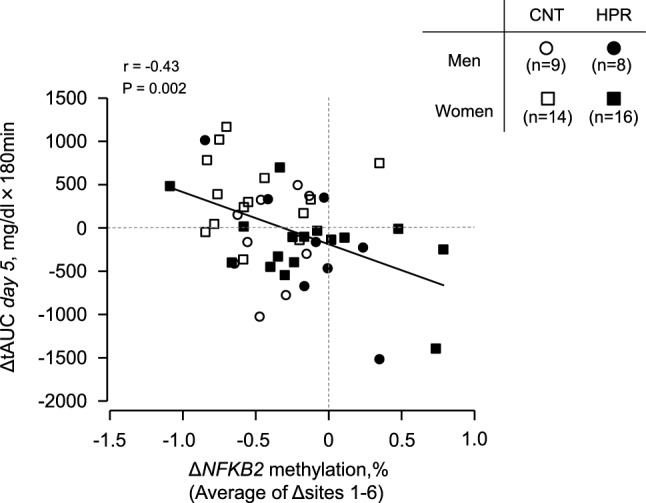
Relationships between the changes in *NFKB2* gene methylation (Δ*NFKB2* methylation) vs. the changes in tAUC at standardized breakfast intake (ΔtAUC *day 5*). Individual values of 23 subjects in the CNT group and 24 subjects in the HPR group. The changes in average *NFKB2* gene methylation values (CpG sites 1–6) are adjusted by ANCOVA with the pre-intervention values as a covariate

We used Pearson’s chi-square test or Fisher’s exact probability test to examine any significant differences in gender distribution, past and current health status, and medication usage by subjects between the CNT and HPR groups (Supplemental Table 1). We used one-way ANOVA to examine any significant differences in training achievements and dietary intake during the intervention (Table 2 and Supplemental Table 3) between the CNT and HPR groups. We also used this model to examine any significant differences in physical characteristics, blood constituents, [Glc] by CGM, and the methylation of the *NFKB2* gene before the intervention (Table 1 and Supplemental Table 4), as well as their changes after the intervention (Table 1, Figs. 3B, 4 and Supplemental Table 4) between the CNT and HPR groups. We used two-way [group x time] ANOVA for repeated measures to examine any significant differences in these variables before vs. after the intervention (Table 1, Figs. 3B, 4 and Supplemental Table 4). Moreover, we examined any significant differences in their changes after the intervention between the groups by ANCOVA using the pre-intervention values for each subject as covariates (Table 1, Figs. 3B, and 4), and the values after the adjustment by ANCOVA are presented where appropriate (Figs. 4 and 5) [24]. We examined any significant effects of intervention (time) on a transient [Glc] response to breakfast intake by two-way ANOVA [time (before vs. after the intervention) x time since starting breakfast intake] for repeated measures (Fig. 3A). We examined any significant effects of group on the transient [Glc] response before the intervention and their changes after the intervention by two-way ANOVA [group x time since starting breakfast intake] for repeated measures (Fig. 3A). We used the Tukey–Kramer test as a subsequent post hoc test for any pairwise comparisons. We used Pearson’s correlation coefficient to examine any significant correlations between the changes in the methylation of the *NFKB2* gene vs. those in HbA1c and tAUC on *day 5* by CGM (Fig. 5). P values < 0.05 were considered significant. The values were expressed as the mean ± standard error (SE) unless otherwise indicated.

## Results

There were no significant differences in physical characteristics, blood constituents, [Glc] by CGM, past and current health status, medication usage by subjects, and methylation of the *NFKB2* gene before the intervention (Table 1, Fig. 3A and Supplemental Table 1 and 4) between the CNT and HPR groups (all, P > 0.09). We confirmed no significant difference in gender distribution of the subjects for the analyses between the groups (all, P > 0.7). In addition, there were no significant differences in training achievements (Table 2) or dietary intake (Supplemental Table 3) during the intervention between the groups (all, P > 0.4). However, after the intervention, as shown in Table 1, [Glc]_fast_ significantly increased in the CNT group (P = 0.004) while not in the HPR group (P > 0.3). Also, overall mean [Glc], TIR and TAR by CGM tended to improve more in the HPR group than in the CNT group, but with no significant difference between the groups (P > 0.15). However, we found significantly greater reductions in pre-breakfast SD (P = 0.013) and post-breakfast tAUC (P = 0.008) by CGM in the HPR group than in the CNT group. Body weight, body mass index, and fasting insulin concentrations significantly increased after the intervention in both groups (all, P < 0.01), but we found no significant differences in the increases between the groups (all, P > 0.5). Systolic blood pressure increased only in the CNT group (P = 0.034) with no significant difference between the groups (P > 0.5). Other variables remained unchanged after the intervention in both groups (P > 0.1). Moreover, all significant interactive effects of [group x time] by two-way ANOVA in Table 1 were confirmed by ANCOVA with the pre-intervention values included as covariates.

Figure 3A shows the trend changes in post-breakfast [Glc] measured by CGM over 180 min when the standardized breakfast was given on *day 5* before and after the intervention in the CNT and HPR groups, respectively*.* As in the figure, although the profile of [Glc] remained unchanged after the intervention in the CNT group, it tended to decrease after the intervention in the HPR group (P = 0.11) with significant greater reductions in the HPR group than in the CNT group from 20 to 130 min from starting breakfast intake (P = 0.034, see the figure legend for further details of statistical results). As a result, we found a significantly greater reduction in tAUC after the intervention in the HPR group than in the CNT group (P = 0.035) (Fig. 3B).

As shown in Supplemental Table 4, we found that methylation at 3 of 6 CpG sites in the promoter region of the *NFKB2* gene decreased significantly in the CNT group (all, P < 0.05) while only at one site in the HPR group (P = 0.007). Then we determined the average changes across CpG sites 1–6 in each group after adjustment with the pre-intervention values by ANCOVA, and we show the results in Fig. 4. As in the figure, we found that the methylation decreased in the CNT group (P = 0.011), while it remained unchanged in the HPR group (P > 0.2), with a significant difference between the groups (P = 0.004).

Figure 5 shows the relationship between the changes in the *NFKB2* gene methylation vs. tAUC when the standardized breakfast was given on *day 5*. The change in the *NFKB2* gene methylation after adjustment with the pre-intervention values by ANCOVA was inversely and significantly correlated with that in tAUC (r = −0.43, P = 0.002) when the data were pooled from 23 subjects in the CNT and 24 subjects in the HPR group. Furthermore, we confirmed that the change in *NFKB2* gene methylation was marginally correlated with the change in HbA1c (r = −0.28, P = 0.055) when the data were pooled from 23 subjects in the CNT group and 25 subjects in the HPR group, though not shown.

## Discussion

The major findings of the present study were that HPP rice intake during 4-month IWT improved glycemic control while it suppressed reduction in the methylation of the *NFKB2* gene, and that there was a significant and inverse correlation between the changes in the *NFKB2* gene methylation and glycemic control in hyperglycemic older people who had performed IWT for ≥ 6 months.

As shown in Table 1, we found that after the intervention, the pre-breakfast SD of [Glc] and post-breakfast tAUC measured by CGM decreased significantly more in the HPR group than in the CNT group when averaged for 4 days. On the other hand, [Glc]_fast_ in a spot sample increased significantly after the intervention only in the CNT group, but the changes in [Glc]_fast_ in a spot sample of blood were not significantly different between the groups.

Matsuzaki et al. [25] examined the effects of HPP rice intake for a year on glycemic control in older people where HPP rice and white rice, 100 g wet weight each per day, were ingested, and they compared the effects with those in the CNT group where 200 g wet weight of white rice was ingested. They found no significantly greater improvements in [Glc]_fast_ and HbA1c in a spot sample of blood after intervention in the HPR group than in the CNT group. One of the possible reasons they saw no significant effects of HPP rice intake on glycemic control was that they did not control subjects’ physical activity prior to and during the intervention. In other words, interindividual variation in physical activity might have masked the effects of HPP rice intake. Another possible reason was that [Glc]_fast_ values in a spot sample of blood varied according to the sampling timing and that HbA1c did not reflect dynamic changes in [Glc], which might have made it difficult to detect any change in glycemic control after intervention. Indeed, in the present study, we found no significant difference in changes in [Glc]_fast_ and HbA1c after the intervention in a spot sample of blood between the CNT and HPR groups. On the other hand, we found significantly greater reductions in the pre-breakfast SD of [Glc] and post-breakfast tAUC in the HPR group than in the CNT group when averaged for 4 days. Thus, it might be necessary to monitor physical activity prior to and during the intervention, as well as [Glc] continuously for a few consecutive days before and after the intervention, in order to detect improvement in glycemic control through HPP rice intake, which is assumed to be less prominent than that brought about through medication. As for the reason for the increase in [Glc]_fast_ in the CNT group, it is likely a result of seasonal change due to adaptation to lower atmospheric temperature, including increased energy intake and subsequent weight gain in winter [26].

As shown in Fig. 3A, after the intervention, the transient increase in [Glc] after breakfast measured by CGM remained unchanged in the CNT group while it tended to be suppressed in the HPR group, with a significantly greater reduction in tAUC in the HPR group (Fig. 3B).

Karstoft et al. [11] examined the effects of 4-month IWT on glycemic control using CGM in type 2 diabetic patients and suggested that mean [Glc] during 48 h for 2 consecutive days deceased after the training with increased V̇O_2peak_ while none of these effects was observed in the sedentary control groups. As for the mechanisms, they suggested that insulin sensitivity in the muscles was enhanced after the training by showing increased insulin-stimulated phosphorylation of AS160 in the sampled muscle tissues [27]. Together, as for their overall mechanisms, we speculated that the deterioration of mitochondrial function with aging generates ROS, injures cells and tissue, and thereby induces chronic inflammatory responses throughout the body. When chronic inflammation occurs in adipose tissue, it promotes insulin resistance and can lead to type 2 diabetes [10].

On the other hand, in the present study, the mechanisms suggested by Karstoft et al. [11] unlikely worked because we found no increase in eV̇O_2peak_ in the HPR group (Table 1). Instead, we propose that anti-oxidant polyphenols contained in the ingested HPP rice likely absorbed ROS generated by mitochondria, suppressed chronic inflammation, and improved glycemic control. Accordingly, in the present study, we measured methylation of the *NFKB2* gene as an index of chronic inflammation in the whole body. The *NFKB2* gene is a member of the *NFKB* family, and a well-known transcriptional regulator that plays a central role in inflammation by inducing pro-inflammatory cytokines synthesis of tumor necrosis factor (TNF)-α, interleukin (IL)−1β, IL-6, and IL-8 [28–30]. In addition, Csiszar et al. [31] have suggested that the *NFKB* gene is a main mediator of inflammation and endothelial dysfunction with aging.

There have been several studies investigating associations between oxidative stress, chronic inflammation, and deteriorated glycemic control [32–36]. They suggested that plasma concentrations of oxidative stress markers were elevated in prediabetic patients [32, 33], that the markers determined cross-sectionally were significantly correlated with [Glc]_fast_, HbA1c, and postprandial [Glc] in prediabetes and diabetic patients [34, 35], and that the markers decreased after intervention with reduced [Glc]_fast_ and improved post-meal tAUC in diabetic patients [36]. These studies indicated that changes in oxidative stress were tightly linked with glycemic control.

As a result, we found in the present study that the methylation of the *NFKB2* gene was reduced in the CNT group while not in the HPR group, with a significant difference in the reduction between the groups (Fig. 4 and Supplemental Table 4). In addition, we found a significant and inverse correlation between the changes in the methylation of the *NFKB2* gene and glycemic control (Fig. 5).

It has been reported that a supplemental polyphenol intake similar to the amount used in the present study improved HbA1c in type 2 diabetic patients [37]. On the other hand, in the present study, because all subjects in the HPR group consumed the same amount and type of rice for testing during the intervention, we could not identify any specific contribution of each phenolic compound to improve the glycemic control with suppressed reduction in the methylation of *NFKB2* in the HPR group. However, ferulic acid is one of the most abundant phenolic compounds in HPP rice (Supplemental Table 2) and a well-known scavenger of ROS [38]; therefore, it could play a major role in improving glycemic control by suppressing *NFKB* activity.

Indeed, there have been a few studies examining the effects of ferulic acid on glycemic control in Streptozotocin-induced diabetic rats [39, 40], and they have suggested that administration of ferulic acid at a dosage of 50 mg/kg, much higher than the 0.8 mg/kg of total phenolic content in the present study, suppressed ROS production, oxidative stress, *NFKB* activity, and *NFKB* dependent pro-inflammatory cytokines TNF-α, IL-1β and IL-6, with lowered [Glc]. In the present study, we found that the methylation of the *NFKB2* gene promoter region was reduced in the CNT group while not in the HPR group, suggesting that the expressions of pro-inflammatory cytokines were suppressed in the HPR group. Thus, our study, which involved human subjects given HPP rice during IWT, confirmed the results previously reported in animal studies [39, 40] using a dosage of total polyphenols suggested to be effective in humans [37].

As noted earlier, HPP rice also contains more GABA than does white rice (Table 3). In a study involving middle-aged and older people with obesity and prediabetes, de Bie et al. [41] investigated whether oral intake of 500 mg/day of GABA might improve postprandial glucose response but found that it did not. Thus, it is unlikely that the much smaller GABA intake (10 mg/day) from HPP rice in the present study had any significant effect on glycemic control. Similarly, as shown in Supplemental Table 2, HPP rice contains a greater amount of free amino acids compared to white rice. However, to our knowledge, there are no studies suggesting effects of such a small amount of amino acid intake on glycemic control. Furthermore, although not shown in the present study, HPP rice reportedly contains 50 ~ 60% of the B vitamins found in brown rice [9], but this amount would not be enough to improve glycemic control [42–44].

Limitations of this study should be acknowledged. Although we explained the improved glycemic control in the HPR group from the scavenger mechanisms for ROS generated by mitochondria, we could not exclude involvement or change in gut flora, short chain fatty acids, and bile acids in the improvement [45, 46]. Further studies are needed to address these deep mechanism actions of HPP rice.

In conclusion, HPP rice intake during IWT improved glycemic control with suppressed reduction in the methylation of the *NFKB2* gene in hyperglycemic older people.

## Supplementary Information

Below is the link to the electronic supplementary material.

Supplementary file1 (DOCX 37 KB)

## Data Availability

The datasets generated and/or analyzed during the present study are not publicly available due to security issues but are available from the corresponding author upon reasonable request.
