# Supplementary material for: Effects of high-pressure-processed rice intake during interval walking training on glycemic control and NFKB2 gene methylation in hyperglycemic older people
Source: Eur J Nutr. 2024 Nov 26;64(1):26. doi: 10.1007/s00394-024-03536-2 (PMC11599310; doi:10.1007/s00394-024-03536-2)
Supplement: Supplementary file 1 — Supplementary file1 (DOCX 37 KB) [file 394_2024_3536_MOESM1_ESM.docx]

**Supplemental Table 1: Past and current health status and medication usage of subjects**

|  | CNT |  | HPR |  |
| --- | --- | --- | --- | --- |
| n | 24 |  | 25 |  |
| Sex (men: women) | 9 : 15 |  | 8 : 17 |  |
| Physical activity | 2.0 ± 0.0 |  | 2.0 ± 0.0 |  |
| Current alcohol consumers, %† | 50.0 |  | 36.0 |  |
| Current smokers, % | 4.2 |  | 8.0 |  |
|  |  |  |  |  |
| Anamnesis, % |  |  |  |  |
| Diabetes mellitus†† | 20.8 |  | 28.0 |  |
| Hypertension | 54.2 |  | 52.0 |  |
| Hyperlipidemia | 29.2 |  | 36.0 |  |
| Obesity | 25.0 |  | 24.0 |  |
|  |  |  |  |  |
| Current medication usage rate, % |  |  |  |  |
| For diabetes mellitus | 12.5 |  | 28.0 |  |
| For hypertension | 54.2 |  | 52.0 |  |
| For hyperlipidemia | 29.2 |  | 36.0 |  |

Values are mean ± SE or % number of subjects. From answers to questionnaire given to subjects before participating in the present study. Physical activity level was scored as 1 = light, 2 = moderate, or 3 = high, according to physical activity and energy requirement guidelines [1]. †Alcohol consumer indicates alcohol intake >10 g/day. ††Type 2 diabetes only, not including type 1.

**Reference**

1. Ministry of Health, Labor and Welfare of Japan (2010) “Energy”. In: Dietary Reference Intakes for Japanese 2010. Daiichi Shuppan, Tokyo, pp 43–61 (in Japanese)

**Supplemental Table 2:** **Contents of individual** **phenolic acids and free amino acids of the cooked test rice per daily intake**

|  | | White rice | HPP rice |
| --- | --- | --- | --- |
| Phenolic acids, mg† | |  |  |
|  | Ferulic acid | 0.26 | 1.06 |
|  | Sinapic acid | 0.06 | 0.98 |
|  | *p*-Coumaric acid | 0.10 | 0.38 |
|  | *p*-Hydroxybenzoic acid | 0.08 | 0.16 |
|  | Vanillic acid | 0.08 | 0.10 |
|  | Syringic acid | 0.02 | 0.02 |
|  |  |  |  |
| Free amino acids, mg†† | |  |  |
|  | Asparagine | 5.10 | 15.24 |
|  | Arginine | 5.58 | 14.28 |
|  | GABA | 0.62 | 10.30 |
|  | Glutamic acid | 6.02 | 8.76 |
|  | Aspartic acid | 4.54 | 7.04 |
|  | Serine | 0.60 | 5.46 |
|  | Leucine | 0.08 | 4.76 |
|  | Valine | 0.08 | 4.26 |
|  | Tyrosine | 0.08 | 3.62 |
|  | Proline | 0.20 | 3.12 |
|  | Phenylalanine | 0.08 | 3.10 |
|  | Methionine | 0.32 | 2.64 |
|  | Histidine | 0.32 | 2.46 |
|  | Isoleucine | 0.02 | 2.32 |
|  | Threonine | 0.06 | 2.22 |
|  | Lysine | 0.20 | 2.10 |
|  | Glutamine | 0.10 | 1.98 |
|  | Tryptophan | 0.02 | 1.56 |
|  | Alanine | 0.08 | 1.50 |
|  | Glycine | ND | ND |
|  | Cysteine | ND | ND |

The values show the amounts of the phenolic acids and free amino acids of cooked test rice per day (150 g dry weight before cooked) which was pre-packaged into individual servings. HPP rice, high-pressure-processed rice; GABA, γ-amino butyric acid; ND, not determined. The data in this table are, with permission, from [1]. †Soluble phenolic compounds in white rice and HPP rice flour were extracted, respectively, with 50% acetone/H_2_O for determination of total phenolic content by the Folin-Ciocalteu method using ferulic acid equivalents instead of garlic acid (**Table 3**). The rice extracts were then hydrolyzed by aq.NaOH to be formed of free phenolic acids for determination of each phenolic acid content using HPLC. ††Free amino acids in white rice and HPP rice flour were extracted, respectively, with 0.02 M HCl for determination of free amino acid contents using LC/MS.

**Reference**

1. Maekawa A (2020) Functional food ingredients of high-pressure processed rice and bioregulatory functions in humans (in Japanese). Master thesis for Shinshu University Graduate School of Science and Technology.

**Supplemental Table 3: Dietary intake per day**

|  | CNT |  | HPR |  |
| --- | --- | --- | --- | --- |
| n | 24 |  | 24 |  |
| Sex (men: women) | 9 : 15 |  | 7 : 17 |  |
| Energy, kcal | 2028 ± 72 |  | 2005 ± 62 |  |
| Protein, g | 76 ± 3 |  | 77 ± 3 |  |
| Fat, g | 61 ± 3 |  | 62 ± 3 |  |
| Carbohydrate, g | 267 ± 8 |  | 270 ± 7 |  |
| Sodium, g | 10.0 ± 0.6 |  | 9.8 ± 0.5 |  |

Values are the mean ± SE. We analyzed 24 subjects for both groups since we failed in the measurement for one man in the HPR group. The dietary survey was conducted for 7 days during both pre- and post-intervention assessment periods, and only the result of post-intervention assessment is shown in the table. The nutritional intake values include the rice for testing. There were no significant differences in any variables between the groups (P>0.6). Also, we confirmed no significant differences in any variables at baseline and the changes after the intervention between the groups (P>0.06).

**Supplemental Table 4: Methylation of the *NFKB2* gene promoter region at baseline and changes after intervention assessed by pyrosequencing**

|  | Baseline | | |  |  | Changes after intervention | | | | Two-way ANOVA  [group x time]† |
| --- | --- | --- | --- | --- | --- | --- | --- | --- | --- | --- |
|  | CNT |  | HPR | |  | CNT |  | HPR |  | P value |
| n 23 25 | | | | |  | 23 |  | 25 |  |  |
| Sex (men: women) 9 : 14 8 : 17 | | | | |  | 9 : 14 |  | 8 : 17 |  |  |
| *NFKB2* gene methylation, % cytosine methylated | | | | |  |  |  |  |  |  |
| CpG site 1 | 2.15 ± 0.16 |  | 2.31 ± 0.19 | |  | -0.29 ± 0.17 |  | -0.17 ± 0.17 |  | 0.62 |
| site 2 | 2.69 ± 0.29 |  | 2.79 ± 0.24 | |  | -0.35 ± 0.19* |  | -0.25 ± 0.22 |  | 0.74 |
| site 3 | 2.12 ± 0.23 |  | 2.32 ± 0.17 | |  | -0.41 ± 0.24 |  | -0.08 ± 0.17 |  | 0.26 |
| site 4 | 2.67 ± 0.22 |  | 3.00 ± 0.18 | |  | -0.39±0.16** |  | -0.30± 0.18** |  | 0.72 |
| site 5 | 3.43 ± 0.29 |  | 3.52 ± 0.28 | |  | -0.65 ± 0.24* |  | -0.13 ± 0.19 |  | 0.09 |
| site 6 | 1.86 ± 0.17 |  | 1.75 ± 0.12 | |  | -0.39 ± 0.20 |  | 0.00 ± 0.12 |  | 0.10 |

Values are the mean ± SE. The target region of the *NFKB2* was located -1238 to -1206 upstream of the transcription start site. †Interactive effect of group x time (before vs. after intervention). Significant differences from pre-intervention value, *P<0.05 and **P<0.01.

.
